# Novel moisturized and antimicrobial hand gel based on zinc-aminoclay and *Opuntia humifusa* extract

**DOI:** 10.1038/s41598-021-97363-8

**Published:** 2021-09-08

**Authors:** Hien Thi Hoang, Vinh Van Tran, Vu Khac Hoang Bui, Oh-Hyeok Kwon, Ju-Young Moon, Young-Chul Lee

**Affiliations:** 1 Department of BioNano Technology, Gachon University, 1342 Seongnam-daero, Sujeong-gu, Seongnam-si, Gyeonggi-do 13120 Republic of Korea; 2 Department of Beauty Design Management, Hansung University, 116 Samseongyoro-16 gil, Seoul, 02876 Republic of Korea; 3 Well Scientific Laboratory Ltd., 305, 3F, Mega-center, SKnTechnopark, 124, Sagimakgol-ro, Jungwon-gu, Seongnam-si, Gyeonggi-do Korea

**Keywords:** Antimicrobials, Infectious diseases

## Abstract

The high antimicrobial ability and low toxicity of zinc-aminoclay (ZnAC) are claimed in our previous reports. In this study, we formulate a novel hand gel based on ZnAC and *Opuntia humifusa* (*O. humifusa*) extract, which is a high moisturizing agent. The antimicrobial activity, cytotoxicity, moisturizing effect, and clinical skin irritation of the hand gel are evaluated. The hand gel with 0.5 wt.% ZnAC and 1.0 v/v% *O. humifusa* extract can kill more than 99% *Escherichia coli* (gram-negative bacteria) and *Staphylococcus aureus* (gram-positive bacteria) after 24 h. Toxicity evaluation shows that, the hand gel does not affect the viability of mammalian HaCaT cells. Additionally, skin moisture is increased by applying the hand gel while its viscosity is at the standard level of commercial products. The hand gel has a skin irritation index of 0.0 and is classified as a non-irritating product. We successfully formulated hand gel from ZnAC, glucomannan, glycerol, and *O. humifusa* extract. Owing to the high antimicrobial activity and skin protection of hand gels, they are suitable to be used as hand sanitizers in restaurants, hospitals, and homes effectively.

## Introduction

Hands are the essential method of transmission of numerous irresistible infectious, especially among school-matured children^1^. Hand cleanliness is viewed as the most significant and compelling contamination control measure to prevent the transmission of nosocomial microbes in medical services settings^2^. According to the US Centers for Disease Control and Prevention and the Association for Professionals in Infection Control and Epidemiology, basic handwashing is the absolute most significant method for forestalling the spread of infectious diseases^3,4^. Hand washing has for some time been perceived as a significant sterile technique in forestalling the transmission of irresistible illnesses^5,6^. Foodborne outbreak research has found that poor personal hygiene practices by grocers contribute to the transmission of pathogens in many outbreaks^6^. In particular, the world is currently struggling to manage the most widespread pandemic of the century, i.e., Coronavirus Disease 2019 (COVID-19). With no clinically supported medicines accessible, the solitary alternative is to cling to the preventive measures given by the World Health Organization (WHO)^7^. Among many precautions, hand washing with cleaner and water has been underlined the most because of its cost-effectiveness and easy access to the general public^8^.

Owing to the importance of hand sanitizers in combating the Covid-19, their demand has increased significantly in 2020, with a 600 times increase in production^9^. A fundamental component in hand sanitizers is alcohol, whose content must constitute 60% for hand sanitizers to be effective in eliminating pathogens on hands^10^. Alcohol is a drying agent and its effect on the epidermal layer of skin is dehydrationn^11^. In most cases, dry skin is not a serious problem, however, it can lead to dermatological complications when managing an infection, for example. Moreover, skin is a natural barrier to pathogens and hand sanitizers should only be used when necessary, e.g., when hand washing with regular soap is not possible. Otherwise, the good bacteria (microbiome) on the hand may be degenerated, potentially resulting in antibiotic resistance and rendering one more susceptible to allergies. Furthermore, the WHO has recommended guidelines for developing hand sanitizers that include hydrogen peroxide^12^. The concentration of hydrogen peroxide in sanitizers must be controlled owing to its oxidative and corrosive nature. When used in higher concentrations, hydrogen peroxide can cause lipid peroxidation, which results in a chemical burn on the skin surface.

Aminoclays, which have 3-aminopropyl functionalities, can be synthesized simply via the sol–gel method at room temperature using different cationic salts and organotrialkoxysilanes as precursors. Owing to the protonation of amine groups in aqueous solutions, aminoclays are free and reversibly delaminated in water. With the infusion of less polar solvents like ethanol, they can be effectively returned to precipitate form^13^. In both biological and environmental applications, aminoclays can interact with the negatively charged lipid membrane of bacteria/algae via electrostatic interaction^13^.

Recently, many researchers have reported the antimicrobial effects of nanoclay composite films that were arranged utilizing organically modified nanoclays. It was hypothesized that these properties might be due to the quaternary ammonium (–NH_3_^+^) groups of organically modified clay in an aqueous solution^14^. Utilizing organically modified clay is better than clay-based inorganic antibacterial clays, which use heavy metals, as the latter can result in serious metal toxicity in people and the environment. Furthermore, these heavy metals decline the antibacterial activity because of the formation of insoluble compounds at the neutral pH^15,16^. Therefore, the clays reported hitherto are too insoluble, which limits their applications. To the best of our knowledge, studies regarding the synthesis and antimicrobial activity of zinc-aminoclay (ZnAC) as well as the evaluation of ZnAC for antimicrobial activity against bacteria, fungi, and multidrug-resistant strains. It has been investigated that the mechanism of antimicrobial action is dependent on membrane depolarization, the release of intracellular enzymes, and scanning electron microscopy. It was discovered that dispersed ZnAC may apply for antimicrobial activity through membrane interruption.

The genus *Opuntia* (*Cactaceae*) includes about 2300 species and is native to the Americas^17,18^. Melgar et al. (2017) showed the evidences to determine *Opuntia *spp. were fully characterized regarding their phenolic and betalain composition and correlated with their antioxidant and antimicrobial activities^19^. Therefore, *Opuntia* species have been used widely as ornamental plants, foodstuffs, forage crops, and as medicinal plants in arid areas of the world^20–22^. Among biomedical plants, *Opuntia humifusa (O. humifusa)*, also known as Korean Cheonnyuncho, is a member of the *Cactaceae* family widely distributed in the southern regions of the Korean peninsula; it is rich in nutrients (i.e., betalains, vitamins, minerals, phenolic compounds, flavonoid compounds, and polysaccharides) contains high concentrations of total polyphenols and flavonoids compared to other cactus species^23^. Therein, the phenolic acids include vanillic acid, p-coumaric acid, ferulic acid, syringic acid, 4-hydroxybenzoic acid, protocatechuic acid, salicylic acid, caffeic acid, gallic acid, and sinnapic acid, whereas the flavonoid compounds include rutin, narcissi, kaemferol, and quercetin^24–26^. Based on *O. humifusa’s* outstanding features (e.g., high moisturization, high antioxidant activity, anti-inflammation, anticancer, and anti-diabetes), its extract has been applied extensively for topical-cosmetic, biomedical, and food-additive purposes^27^. *O. humifusa* shows different biological activities, including promoting anti-atherosclerosis^28^, anticancer^25,29^, antibacterial^30^, antioxidant^31,32^, anti-inflammatory^33^, hypoglycemic and hypolipidemic activity^28^, and an alternative treatment of postmenopausal osteoporosis^34^. Accordingly, it has been used for the treatment of various diseases, such as arteriosclerosis, diabetes mellitus, gastritis and hyperglycaemia^25^.

Recently, numerous studies have demonstrated that *O. humifusa* extracts (OHE) can potentially be utilized as an ingredient for skincare application. For example, *O. humifusa* has been shown to exert preventative effects on chemical carcinogenesis in the skin of mice, and its preventive effects have been associated with a reduction in the enzyme detoxification system phase II^35^. Ha et al*.* (2006) indicated that *O. humifusa* extracts (OHE) effectively inhibited the transcription factor associated with microphthalmia, reduced-matrix metalloproteinase-1 (MMP-1), and phosphorylation of c-Jun N-terminal kinase (JNKs). It has been reported that they may as impart whitening and wrinkle-improvement effects in various functional cosmetics^26^. In another study, Park et al*.* (2017) showed the evidence to determine the skincare capability of OHE, including its capacity to regulate ultraviolet B (UVB)-induced hyaluronic acid (HA) production in human cell cultures and hairless mice^27^. This study proved that OHE can significantly stimulate hyaluronic acid synthases (HAS) expression to regulate HA synthesis but lower expression of hyaluronidase (HYAL) transcripts in photo-damaged human keratinocytes. OHE treatments were also found to inhibit the compensatory increases of HA, HABP, and CD44 protein expression in UV-irradiated hairless mice, and to suppress the transepidermal water loss and erythema formation. It suggests that OHE protects the skin from the harmful effects of UVB rays. Consequently, because of *O. humifusa*’s extraordinary highlights, its extract has been applied widely for biomedical, topical-cosmetic, and food-added substance purposes.

In our study, a gel hand based on aminoclay and *O. humifusa* extract was prepared. We combined aminoclays, which offers antimicrobial properties, and *O. humifusa* extract to protect hand skin, thereby reducing dryness caused by frequent hand washing or hand washing using detergent. We evaluated the antimicrobial activity, cytotoxicity, moisturizing effect, and clinical irritation evaluation of the gel hand. The aim of this study is to determine the potential benefit of aminoclay, and *O. humifusa* in protecting hand, and pharmacology.

## Results and discussion

### Viscosity of hand gel formulations

The viscosity of personal care or cosmetics products is one of the most important factors in controlling quality and ensuring customer satisfaction. Furthermore, viscosity is typically used as a parameter to determine the suitable formulations, as it has significantly affected some crucial characteristics such as skin feel and spread-ability but might also affect the skin penetration of incorporated active agents^36^. Moreover, a reasonable viscosity of hand gel formulations is beneficial for the enhancement of its moistening effect on the skin. The measured viscosity values of five hand gel formulations are presented in Fig. S1. The results indicated that the viscosity of the hand gel formulations was between 1400 and 1500 mPa·S, indicating that the hand gels are easily spreadable on the human skin. Moreover, it has been indicated that the addition of ZnAC slightly decreased the viscosity of hand gels, although the viscosity values were still within those of gel products. It has been indicated that glucomannan has an extraordinary capability of absorbing water due to a molecular weight from 200 to 2000 kDa and its solution is very high viscosity^37^. Thus, it is supposed that the viscosity of hand gel formulations is mainly contributed by the presence of glucomannan. However, the interaction between ZnAC and glucomannan through hydrogen bonds of –NH_2_ and –OH functional groups induced the exclusion of water molecules contacting glucomannan^38^, which resulted in a slight reduction of viscosity. Generally, the viscosity of commercial hand gel is between 1000 and 3500 mPa·S. Therefore, the hand gel formulations in this study are appropriate for the development of cosmetic formulations.

### Cell viability

**Figure 1:**
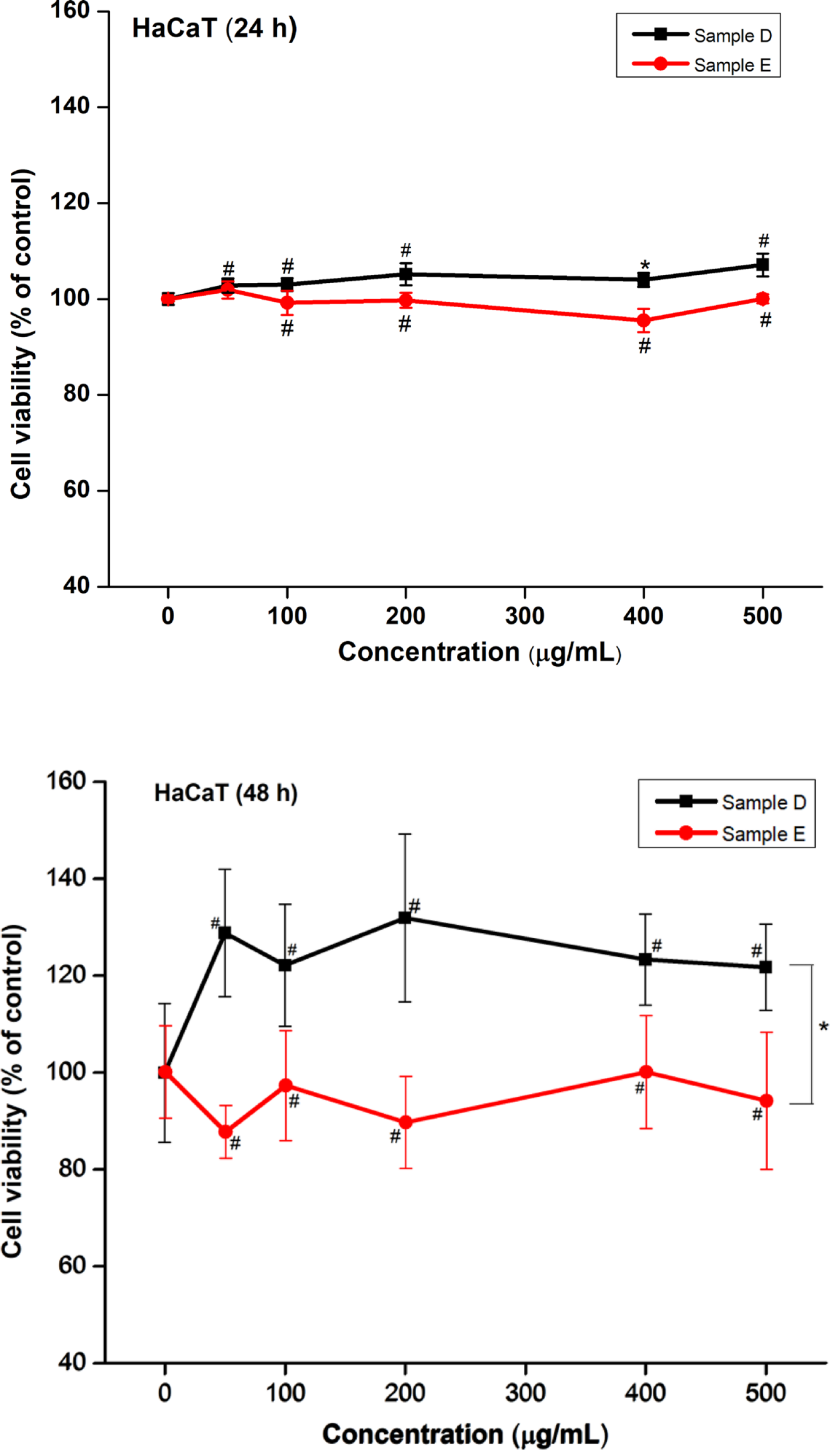
Effect of hand gel on viability of HaCaT after 24 h and 48 h. The results are expressed as mean ± standard error of mean, N = 3, The statistical analysis was performed using ANOVA, p-value more than 0.05 was considered as statistically insignificant. *p < 0.05, ^#^p > 0.05 significant difference vs 0 μg/mL.

First, the biocompatibility of the hand gel formulation was assessed using the MTT assay. The measured cell viability of two hand gel formulations, D and E (Table 1), are presented in Fig. 1. As shown, sample D exhibited negligible toxicity in both high- and low-dose treatments, whereas sample E showed slightly higher toxicity on HaCaT cells after a 24 h treatment. It can be concluded that sample D offered greater biocompatibility than sample E showed on 48 h treatment (*p* > 0.05, α = 0.05).

**Table 1:** The formulations of hand gels in cytotoxicity assay.

Sample	Glucomannan (mg)	ZnAC (mg)	*O. humifusa *(mL)	Glycerol (mL)
D	500	400	1	1
E	500	500	1	1

The results of cell viability assay with different concentrations (0–500 μg/mL) of gel hand on HaCaT cells incubated for 24 h and 48 h were obtained. In the present study, the MTT assay was also used to assess the effectiveness of ZnAC and *O. humifisa* in reducing the effect of ZnAC on HaCaT cells. Briefly, HaCaT cells were pretreated with hand gel (0, 100, 200, 400, and 500 μg/mL) for 24 h and 48 h. Results indicated that viability of HaCaT cells was not affected statistically by any of the treatments using gel hand up to 500 mg/mL for 24 h or 48 h. When the viability was measured 24 h, the hand soap treatment did not show any statistically significant difference (p > 0.05, α = 0.05) compared with the experimental control. The results show that sample D was over 100% in all cases and did not hinder the viability in HaCaT after 24 h and 48 h. Specifically, the survival of HaCaT cells increased with decreasing ZnAC concentrations from 0 to 500 μg/mL. At the lowest concentrations, the hand gel showed the best potential in cell protection cells. Meanwhile, the hand gel treated with *O. humifusa* exhibited an increase in the survival ratio.

### Antimicrobial activity

**Figure 2:**
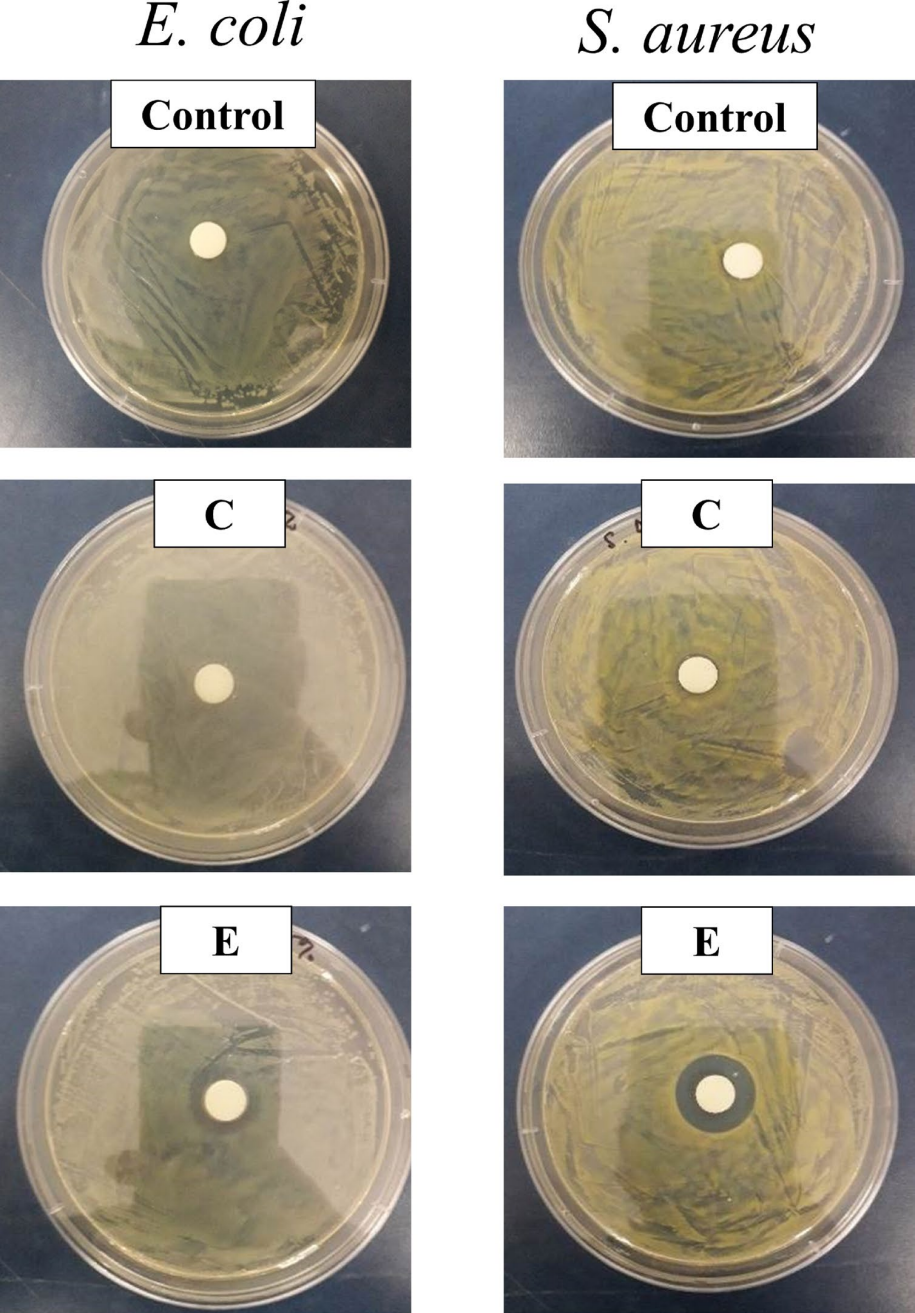
Inhibition zone of hand gels against *E. coli* and *S. aureus.*

**Figure 3:**
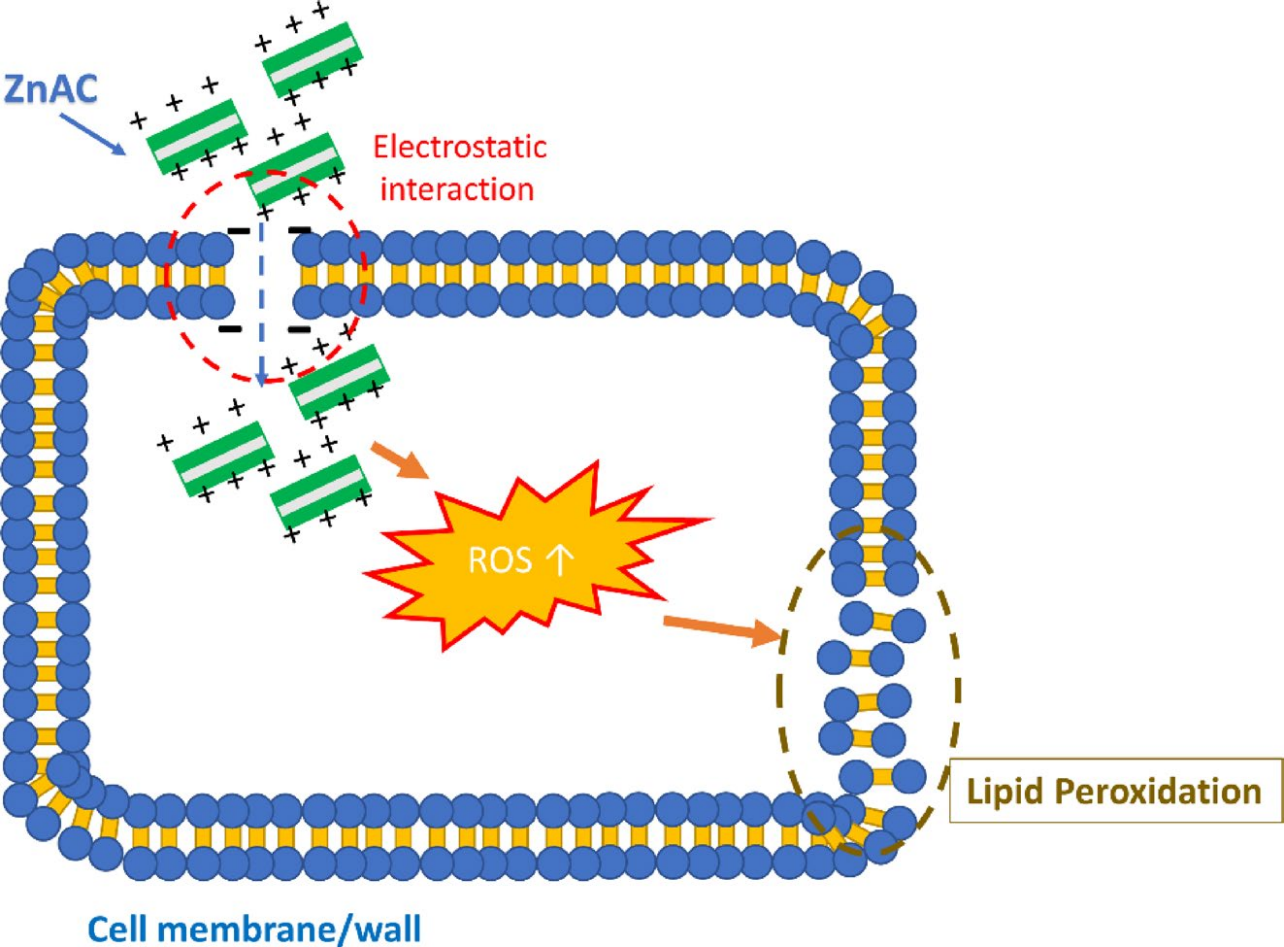
Antimicrobial mechanism of ZnAC. The image was drawn by Microsoft PowerPoint Version 2016.

Based on agar diffusion methods, it can be concluded that the antimicrobial activity of hand gel depends on the concentration of ZnAC in the hand gel formulations. It is believed that *O. humifusa* does not exhibit any antimicrobial activity at 1% (v/v) concentration (Table 2; Fig. 2). For the remaining samples, the only sample with 0.5 wt.% ZnAC showed antimicrobial activity against both *S. aureus* and *E. coli*. It was discovered be noted that the antimicrobial potential of ZnAC against *S. aureus* (Gram-positive) was higher than that against *E. coli* (Gram-negative), which is consistent with our previous report^13^. The differences in the physiology, constitution, and metabolism of the cell and cell wall between the gram-positive and -negative bacteria might have contributed this result^39^. The antimicrobial activity of hand gel formulations with 0.5 wt.% ZnAC was confirmed in the broth microdilution method. The results show that this sample can completely inhibit the growth of both *S. aureus* and *E. coli* after 24 h (Table 3 and Fig. S2). In our previous report, ZnAC showed higher antimicrobial activity compared with magnesium aminoclay (MgAC)^13^. Similar to MgAC, aminopropyl groups (positive charge) of ZnAC can electrostatically interact with the negatively charged lipid membrane of bacteria and disrupt to the lipid bilayer, and membrane permeability, as well as cause the leakage of bacterial content, thereby resulting in thus resulted in the death of bacterial cells. After uptaking by bacterial cells, ZnAC increased the intracellular ROS concentration and significantly increase the production of malondialdehyde (MDA, a lipid peroxidation marker), and lead to the death of cell by the disruption of the bacterial membrane (Fig. 3)^40–42^. Because ZnAC is proved to not release the Zn^2+^ in our previously report, the antimicrobial mechanism via Zn^2+^ release is eliminated^41^.

**Table 2:** Antimicrobial inhibition zone of hand gels.

Sample	Glucomannan (mg)	ZnAC (mg)	*O. humifusa* (mL)	Glycerol (mL)	Inhibition zone (mm)	
					*E. coli*	*S. aureus*
Control	500	0	1	1	0	0
C	500	300	1	1	0	0
E	500	500	1	1	14.9 ± 0.5*	17 ± 0.9*

*The statistical analysis was performed using ANOVA (N = 3), p-value less than 0.05 is considered as statistically significant, *p < 0.05.

**Table 3:** Antimicrobial efficiency of hand gels.

Sample	Bacterial concentration after incubation (CFU/mL)		Antimicrobial efficiency (%)	
	*E. coli*	*S. aureus*	*E. coli*	*S. aureus*
Bacteria suspension (Control)	4.9 ± 2.9 × 10^8^	9.7 ± 2.7 × 10^8^	0	0
E	0	0	> 99.9	> 99.9

In the purpose of evaluating the potential of our hand gels, in reality, we conduct hand spreading experiments. Results indicated that although the initial concentration of total bacteria is deficient (average of 232–270 CFU), bacteria is almost diminished after spread with hand gels. After working for 3 h from the spreading time, the total bacteria in both hands is slightly increased again. However, the number of bacteria in both hands is almost the same (average 25–33 CFU, Fig. S3). Thus, it could be concluded that the anti-microbial performances of alcohol-free ZnAC-based hand gel and alcohol-based commercial hand gel are similar. However, this is a preliminary experiment and should be further improved. More volunteers and complex scenarios should be considered to evaluate the practical applications of ZnAC-base hand gels.

### Skin moisturizing effect

To evaluate the effects of the hand gel formulation on skin hydration, we analyzed the protein levels of HAS-2, HAS-3, and AQP3, in hand-gel-treated HaCaT cells using western blotting. It was discovered that the protein expressions of HAS-2 and HAS-3 increased significantly owing to the hand gel.

Adequate moisturizing ability is vital to the skin’s barrier function^43,44^. The homeostasis of moisture must be maintained for the skin to function appropriately. In addition, water loss from the skin results in aging and damaged skin^45^. Furthermore, skin hydration should be maintained to achieve healthy and youthful skin. Therefore, moisturizing products are vital to the cosmetics industry. Accordingly, with the recent increased interest in naturally derived materials, *O. humifusa* should be considered an effective and safe cosmetic ingredient as it is safely used in medicine and food.

HA produced by HAS is a representative molecule involved in skin moisturizing; therefore, increasing HA synthesis is an effective strategy for improving skin moisturization^46^. Another major factor in skin hydration is AQP3, an aquaporin transmembrane water channel that transports glycerol and water to regulate various physiological functions, such as cellular metabolism and skin hydration. In particular, AQP3 is vital to skin-moisture homeostasis^47,48^. In this study, we analyzed the protein expression of HASs and AQP3 in hand gel with *O. humifusa* treated on HaCaT cells by western blotting. To quantify the protein expression of skin-barrier-function-related factors, HaCaT cells were treated with sample 1, 2, 3, or 4 for 24 h. Subsequently, western blotting was performed to measure protein expression of HAS2, HAS3, and APQ3.

**Figure 4:**
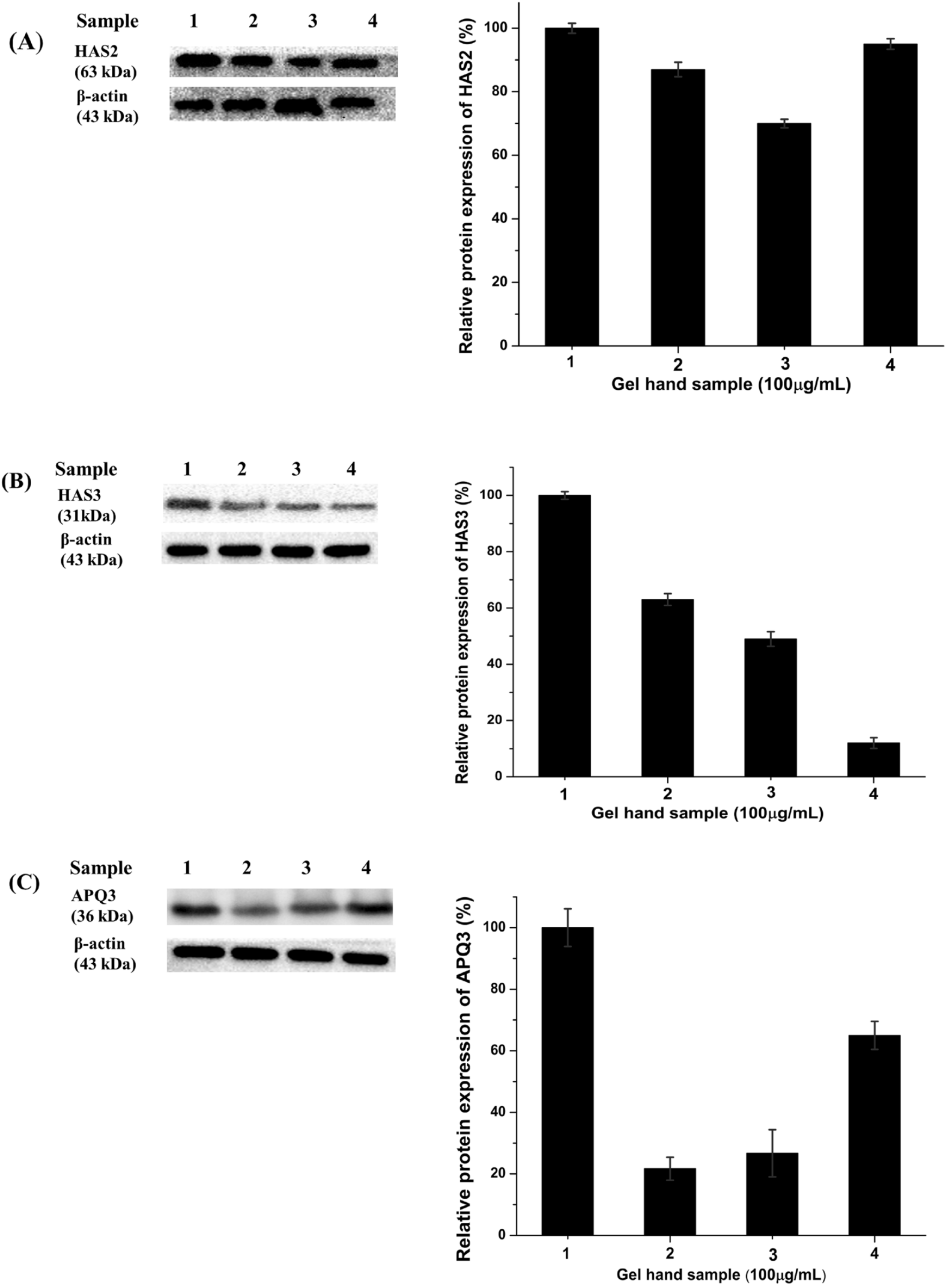
Moisturizing effects of hand gel in HaCaT cells. Expression levels of moisturizing-related proteins [HAS2 (**A**), HAS3 (**B**), and APQ3 (**C**)]. The results are expressed as mean ± standard error of mean, n = 3.

The images of the Western blot analysis showed levels of moisturizing-related proteins (HAS2, HAS3, and APQ3) are presented in Fig. S4. The results show that, *O. humifusa* upregulated the protein of HAS-2, HAS-3, and AQP3. Additionally, sample 1 indicated the highest protein expression on HAS2, HAS3, and AQP3. Therefore, sample 1 can increase HA synthesis, and APQ3 effectively, thereby improving skin moisturization, and skin hydration (Fig. 4).

### Clinical skin irritation evaluation of hand gel

Sample E with 0.5 wt.% ZnAC was chosen for clinical irritation evaluation. Results showed that all candidates have 0 of evaluation grade 1 h and 24 h after removing the patches (Table S1). Therefore, with 0.0 of skin irritation index, sample E was judged as a non-irritating product according to the criteria.

In conclusion, we successfully formulated hand gel from ZnAC, glucomannan, glycerol, and *O. humifusa* extract. Aminopropyl groups (positive charge) of ZnAC were demonstrated to electrostatically interact with the negatively charged lipid membrane of bacteria and disrupted of the lipid bilayer, and membrane permeability, as well as caused the leakage of bacterial content, thereby resulting in in the death of bacterial cells. The hand gel formulations in this study appropriate for the development cosmetic formulations. Furthermore, in vitro experimental results show that the formulations can protect skin hand, as well as offer skin moisturization and hydration. The hand gel has a skin irritation index of 0.0 in clinical irritation evaluation and safe to use as a hand hygiene product. Our results strongly suggested the viability gel hand in promoting hand hygiene, and studies are in progress to confirm our hypothesis. Owing to the high antimicrobial activity and skin protection of hand gels, they are suitable to be used as hand sanitizers in restaurants, hospitals, and homes effectively.

## Methods

### Chemicals and reagents

Zinc chloride (ZnCl_2_; 136.30 g/mol) and 3-aminopropyltrielthoxysilane (APTES, ≥ 98%, 221.37 g/mol), glucomannan (Korea Karagen Co., Ltd), glycerol, cell-proliferation reagent water-soluble tetrazolium salt-1 (WST-1), and trypan blue (0.4%) were purchased from Sigma-Aldrich (St. Louis, MO, USA. Bulk ethanol was obtained from Samchun Pure Chemical (Pyungtack, Korea). *Escherichia coli* ATCC 25922 and *S. aureus* ATCC 25923 bacterial strains were obtained from the culture collection at the Department of Bionano Technology, Gachon University, Korea. *O. humifusa*, known as Korean Cheonnyuncho (grown in Nonsan, Korea), was harvested and washed with water to remove glochids and then stored in a refrigerator. Human keratinocyte cells (HaCaT) were obtained from the Korean Cell Line Bank (Seoul, Korea). DMEM medium (1% Pen/Strep, 1% l-glutamine, and 10% FBS), DPBS buffer, and trypsin/EDTA were obtained from Sigma-Aldrich.

### Preparation of ZnAC and *O. humifusa* extract

In this study, ZnAC was prepared based on a method used in our previous study involving a ZnCl_2_ precursor^41^. Briefly, ZnCl_2_2H_2_O (0.84 g) was completely dissolved in ethanol solution (20 mL), followed a drop-wise addition of APTES (1.3 mL). To form ZnAC, the Zn:Si ratio was adjusted to 1.34. Subsequently, a simple sol–gel reaction occurred and yielded ZnAC in white-slurry form as a resulted of the production of siloxane [Si–O–Si] bonds. After the ZnAC slurry was centrifugated, washed with ethanol, and dried in an oven, ZnAC powder was obtained. According to our previously report, ZnAC has the chemical formula [H_2_N(CH_2_)_3_]_8_Si_8_Zn_6_O_12_(OH)_4_]. It is amorphous aminoclay with worm-like structure and aggregated sheets within 10–100 nm range (average: ~ 54.83 nm; Fig. S5). The zeta potential of ZnAC was + 40.90 mV. More information about ZnAC can be found from our previous articles^13,41^.

*O. humifusa* extract was prepared based on a microwave-assisted process, which is reported in our previous publication^49^. First, a homogeneous mixture of raw *O. humifusa* was obtained after washing and grounding its leaves in a blender. Next, a domestic microwave oven (Magic MMO-20M7) was used to extract *O. humifusa*. The resultant mixture of *O. humifusa* with extremely high viscosity was subjected to microwaves of 800 W power for 10 min. After cooling to room temperature, the brown solid sample was ground and 15 mL of DI water was used to dissolve the active agents. Finally, insoluble impurities were removed and a pure extract solution was obtained through centrifugation and filtering using a 0.22 µm centrifuge tube filter. The supernatant was utilized in the freeze-drying process.

### Preparation of hand gel

The hand gel was prepared by mixing four ingredients including glucomannan, *O. humifusa*, aminoclay, and glycerol according to specific ratios. First, 500 mg of glucomannan was dissolved in 100 mL of DI water under high-speed stirring for 6 h; subsequently, different amounts of ZnAC were added with vigorous stirring. To create a completely homogeneous gel, this mixture was stirred gently for 6 h. Subsequently, 1% (v/v) *O. humifusa* and 1% (v/v) glycerol are inserted to enhance the antimicrobial activity and reduce the sticky of the final gel, which makes a final hand gel product.

In this study, we developed and evaluated five samples of different ratios, as shown in Table 4.

**Table 4:** Different formulations of hand gels investigated in this study.

Sample	Glucomannan (mg)	ZnAC (mg)	*O. humifusa *(mL)	Glycerol (mL)
A	500	100	1	1
B	500	200	1	1
C	500	300	1	1
D	500	400	1	1
E	500	500	1	1

### Viscosity measurement

The viscosity of the hand gel was measured using spindle C50-1, and experiments were performed at 25 °C, and atmospheric pressure, in fixed containers sizes^50^. The spindle speeds were set to 1, 3, 5, 10, and 20 rpm, and the shear rate was to 100 s^−1^ for 60 s. Prior to performing measurements, the hand gel formulations were gently stirred at 25 °C for 2 h, and an equilibrated suspension for 4 h. Glycerol (10%) was utilized as a control sample. The measurement was conducted thrice repeatedly, and the uncertainty of the viscosity values was within 0.01 mPa·s.

### Cell culture and cytotoxicity assay

HaCaT cells were cultured in growth DMEM medium based on a previously reported method^51^. As the density of cells was 70–90% confluent, they were subcultured to achieve the desired density for cytotoxicity and western blotting experiments. For the subculture, the cell culture medium was first removed, and the flask was twice washed with DPBS buffer. To detach the cells from the culture flask, trypsin/EDTA was deposited; subsequently, this cell suspension was then mixed with a new growth DMEM medium in conical tubes. Finally, the cells were collected by centrifuging at 1500 rpm, adjusted to a destiny of 10^5^–10^6^ cells/mL, and incubated consecutively at 37 °C and 5% CO_2_.

In this study, an MTT assay was utilized to evaluate the cytotoxicity of the hand gel formulations. Briefly, the cells with the desired density were seeded and incubated in a transparent 96 well plate at 37 °C and 5% CO_2_ for 24 h. Subsequently, the growth DMEM medium was removed and 100 µL of the hand gel formulations at various concentrations from 0 to 500 µg/mL were added. After 24 h and 48 h, each well was treated with 10 µL of MTT reagent and the plate was incubated for 4 h. Next, each well was 100 µL of solubilization solution, and then incubated overnight. Finally, a microplate reader (Multi-label plate reader, PerkinElmer, Boston, MA, USA) was used to measure the absorbance of the treated cell suspension at a wavelength of 590 nm.

### Western blotting

In this study, to evaluate the skin moisturizing effect of hand gel formulations, we measured the protein expression of hyaluronic acid syntheses (HASs) and aquaporin 3 (AQP3) in HaCaT cells treated by an E formulation using western blotting, which has been described previously^52^. Briefly, HaCaT cells were seeded in 96 well plates and treated with the E formulation for 24 h. Next, total cell lysates were prepared via a lysis buffer and subjected to SDS-polyacrylamide gel electrophoresis and transferred onto polyvinylidene fluoride membranes. The blots were cut prior to hybridization with antibodies during blotting. The total moisturizing-related proteins including HAS-2, HAS-3, and AQP3 were determined based on specific antibodies and visualized using chemiluminescence reagents. Four samples (M1–M4), as listed in Table S2, were used as negative controls.

### Antimicrobial assay

The contaminations of harmful bacteria are one of the most challenging healthcare throughout the world^53^. The antimicrobial activities of the hand gel formulations were evaluated using two methods: (1) agar diffusion^54^, which is a typical test to assess antimicrobial activity by diffusing the compounds in a water-containing agar plate, and (2) broth microdilution^55^. In this study, *Escherichia coli* (*E. coli*, gram-negative bacteria) and *Staphylococcus aureus* (*S. aureus*, gram-positive bacteria) were selected as the model bacteria. *E. coli* and *S. aureus* are two major species that cause different diseases such as diarrhea, anemia, skin infections, orbital cellulitis, urinary tract infections, and eventually death^56^.

Luria Bertani (LB) was used as a medium for the growth of *E. coli* and *S. aureus.* For the agar diffusion test, For the agar diffusion test, the sterilized nutrient agar was poured out Petri dish, and 0.1 mL of each bacterial strain (which was previously adjusted to 10^6^ CFU/mL) was spread on nutrient agar using a loopful. 6-mm diameter sterile disks (Sigma-Aldrich, St. Louis, MO, USA) were impregnated with 50 µL of various hand gel formulations and place on the above culture media. After 72 h incubation at 37 °C, the inhibition zone was determined^57^. In the agar diffusion test, two hand gel formulations (C, E) were selected to evaluate the antimicrobial activity.

Based on the results of the agar diffusion test, the hand gel formulation with the highest antimicrobial activity was selected for the broth microdilution method^55^. In this method, 50 μL of bacterial suspension (~ 10^6^ CFU/mL) was inserted into the wells of a sterile 96 well plate that containing 50 μL of the samples and incubated at 37 °C for 24 h. Control plates were prepared using the culture medium, bacterial suspension only, and DI water. After 24 h, 100 µL of serially diluted broth sample was cultivated on LB agar plates. After cultivation for 72 h, the survival of bacteria was counted.

For the hand spreading experiment, volunteers are recommended not to use any hand sanitizers 24 h before the test and print both of his/her hands on agar plates (for total bacteria) for 5 s^58,59^. Then the volunteers are allowed to spread each hand with optimized ZnAC-based or alcohol-based commercial hand gel (NARD Hand Sanitizer, Breathe-B International Ltd., Daegu, Korea). According to Fouad et al*.* (2018), there are no differences between the dominant hand and non-dominant hands regarding the type or frequency of microbial growth^59^. After working for 3 h, both hands of the volunteers were printed on agar plates again. The agar plates were incubated at 37 °C for 24 h to read the results.

### Clinical skin irritation evaluation of hand gel

The skin irritation was conducted by KC Skin Clinical Research Center Co., Ltd. (Seoul, Korea) in accordance with the Human Body Application Test Management Standard (Good Clinical Practice) and the Helsinki Declaration. The experiment was entrusted by the Korea Ministry of Food and Drug Safety in accordance with Test Method Guidelines for Demonstration of Cosmetic Label Advertisement (2018/03), Guidelines for Human Body Applications Testing and Efficacy Testing of Cosmetics (2017/05), and Guidelines for Evaluating New Raw Materials for Cosmetics (2012/05). The clinical experiment had an Institutional Review Board (IRB) approval number (1-70005235-A-N-01-202006-HR-KC-200701-S1-02) issued by KC Skin Clinical Research Center. A total of 33 adult women (average age: 46.485 ± 7.938 years old) were selected for the test. Suitable candidates are healthy persons without major and chronic physical diseases, including skin diseases. Informed consent was obtained from all participants. 25 μL of hand gel was dropwise added to IQ Ultimate patches (1 cm × 1 cm; Chemotechnique, Vellinge, Sweden) and attached to the skin of candidates. The patches were attached for 24 h. After 24 h, the patches were removed, and the degree of irritation was observed in the next 1 h and 24 h by two experts according to the criteria of the International Contact Dermatitis Research Group (ICDRG). The evaluation standard was presented in Table S3.

Skin irradiation index (Table S4) is calculated as the following equation:

$$ Skin \;irradiation\; index = \Sigma \frac{Grade \times No.\; of\; Responders \times 100}{{4 (Maximum \;grade) \times n (Total \;subjects)}} \times \frac{1}{2}(No. \;of \;evaluation). $$

### Ethics approval

The Clinical skin irritation evaluation was entrusted by the Korea Ministry of Food and Drug Safety and had an Institutional Review Board (IRB) approval number (1-70005235-A-N-01-202006-HR-KC-200701-S1-02) issued by KC Skin Clinical Research Center.

### Consent to participate

Candidates who were selected for the Clinical skin irritation evaluation have notified the purpose, contents of the test, and wrote a consent form for participation in the trial.

## Supplementary Information

Supplementary Information.

## Data Availability

The datasets used and/or analyzed during the current study are available from the corresponding author upon reasonable request.
