# Supplementary material for: Novel moisturized and antimicrobial hand gel based on zinc-aminoclay and Opuntia humifusa extract
Source: Sci Rep. 2021 Sep 8;11:17821. doi: 10.1038/s41598-021-97363-8 (PMC8426495; doi:10.1038/s41598-021-97363-8)
Supplement: Supplementary file 1 — Supplementary Information. [file 41598_2021_97363_MOESM1_ESM.docx]

**Novel moisturized and antimicrobial hand gel based on zinc-aminoclay and *Opuntia humifusa* extract**

Hien Thi Hoang^a,§^, Vinh Van Tran^a,§^, Vu Khac Hoang Bui^a,§^, Oh-Hyeok Kwon^b^, Ju-Young Moon^b*^, Young-Chul Lee^a,c*^

^a^Department of BioNano Technology, Gachon University, 1342 Seongnam-Daero, Sujeong-Gu, Seongnam-Si, Gyeonggi-do 13120, Republic of Korea

^b^Department of Beauty Design Management, Hansung University, 116 Samseongyoro-16gil, Seoul 02876, Republic of Korea

^c^Well Scientific Laboratory Ltd., 305, 3F, Mega-center, SKnTechnopark, 124, Sagimakgol-ro, Jungwon-gu, Seongnam-si, Gyeonggi-do, Korea

**Correspondence:** bora7033@naver.com (J.-Y. Moon), dreamdbs@gachon.ac.kr (Y.-C. Lee)

^§^Equally contributing authors

**Table S1.** Results of skin irritation determination

| **Sample** | **Evaluation grade (Persons)** | | | | | | | | | | **Skin irradiation index** |
| --- | --- | --- | --- | --- | --- | --- | --- | --- | --- | --- | --- |
|  | **1 h after removing patches** | | | | | **24 h after removing patches** | | | | |  |
|  | **0** | **1** | **2** | **3** | **4** | **0** | **1** | **2** | **3** | **4** |  |
| E | 33 | - | - | - | - | 33 | - | - | - | - | 0.0 |

**Table S2**. List of samples for evaluating skin moisturizing effect

| **Sample** | **ZnAC** | **Glucomannan** | ***O. humifusa Extract*** | **DI water** |
| --- | --- | --- | --- | --- |
| 1 | 500 mg | 500 mg | 1 mL | 101 mL |
| 2 | 500 mg | DI water | 1 mL | 101 mL |
| 3 | 500 mg | DI water | DI water | 101 mL |
| 4 | DI water | DI water | 1 mL | 101 mL |

**Table S3.** Skin irritation evaluation standard

| **Sign** | **Grade** | **Criteria** |
| --- | --- | --- |
| - | 0 | Negative |
| + | 1 | Slight erythema, either spotty of diffuse |
| ++ | 2 | Moderate uniform erythema |
| +++ | 3 | Intense erythema a with edema |
| ++++ | 4 | Intense erythema with edema and vesicles |

**Table S4.** Skin irradiation index and its division

| **Skin irradiation index** | **Division** |
| --- | --- |
| 0.0 – 0.9 | No irritation |
| 1.0 – 2.9 | Light stimulation |
| 3.0 – 4.9 | Heavy stimulation |
| Above 5.0 | Strong stimulation |

**
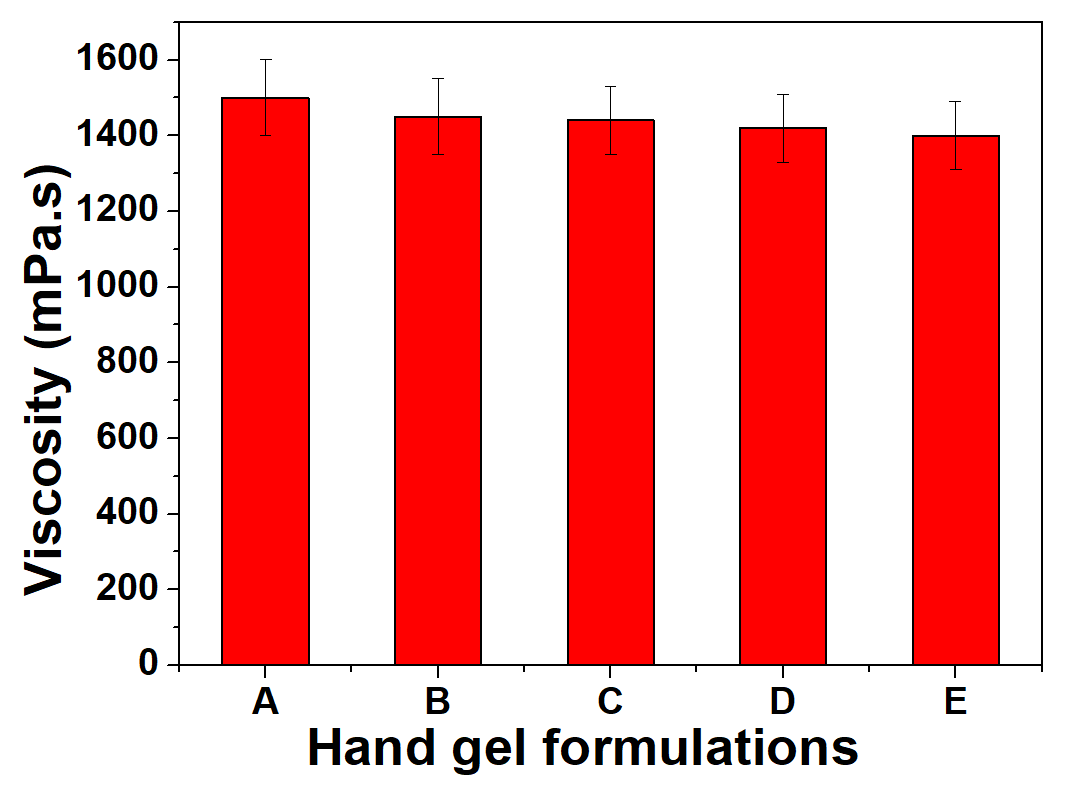
**

**Fig. S1.** Viscosity values of hand gel formulations

**
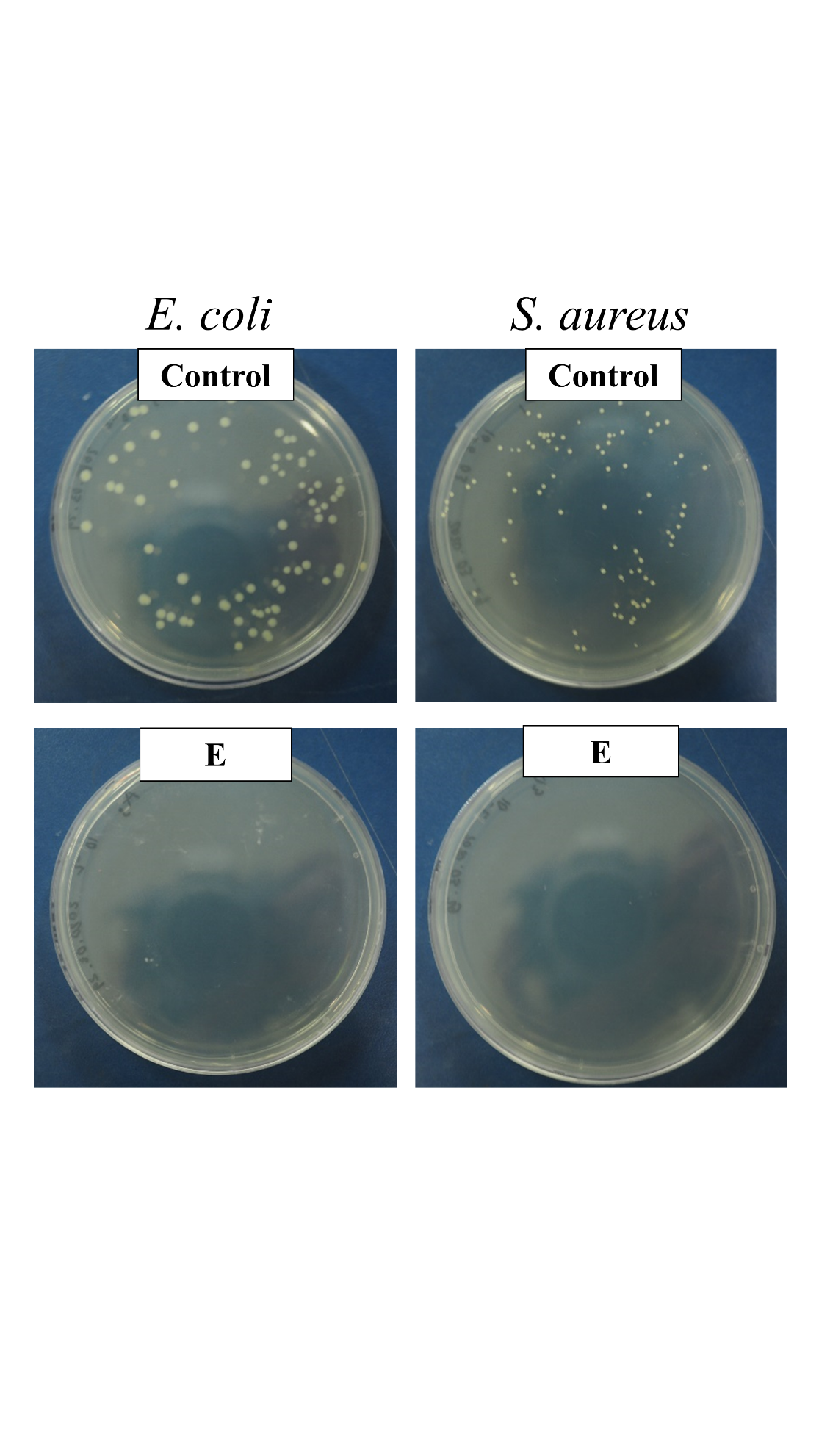
**

**Fig. S2.** Antimicrobial efficiency of hand gels (E)


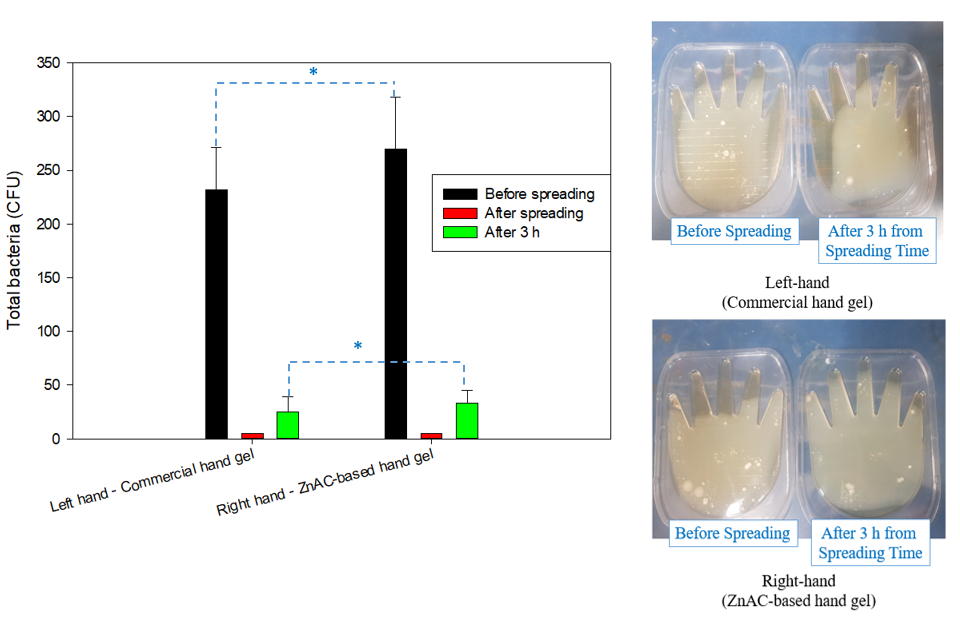


**Fig. S3.** Results of hand spreading experiment. *The statistical analysis was performed using ANOVA (N = 3), ^*^p-value > 0.05: no significant difference*

**
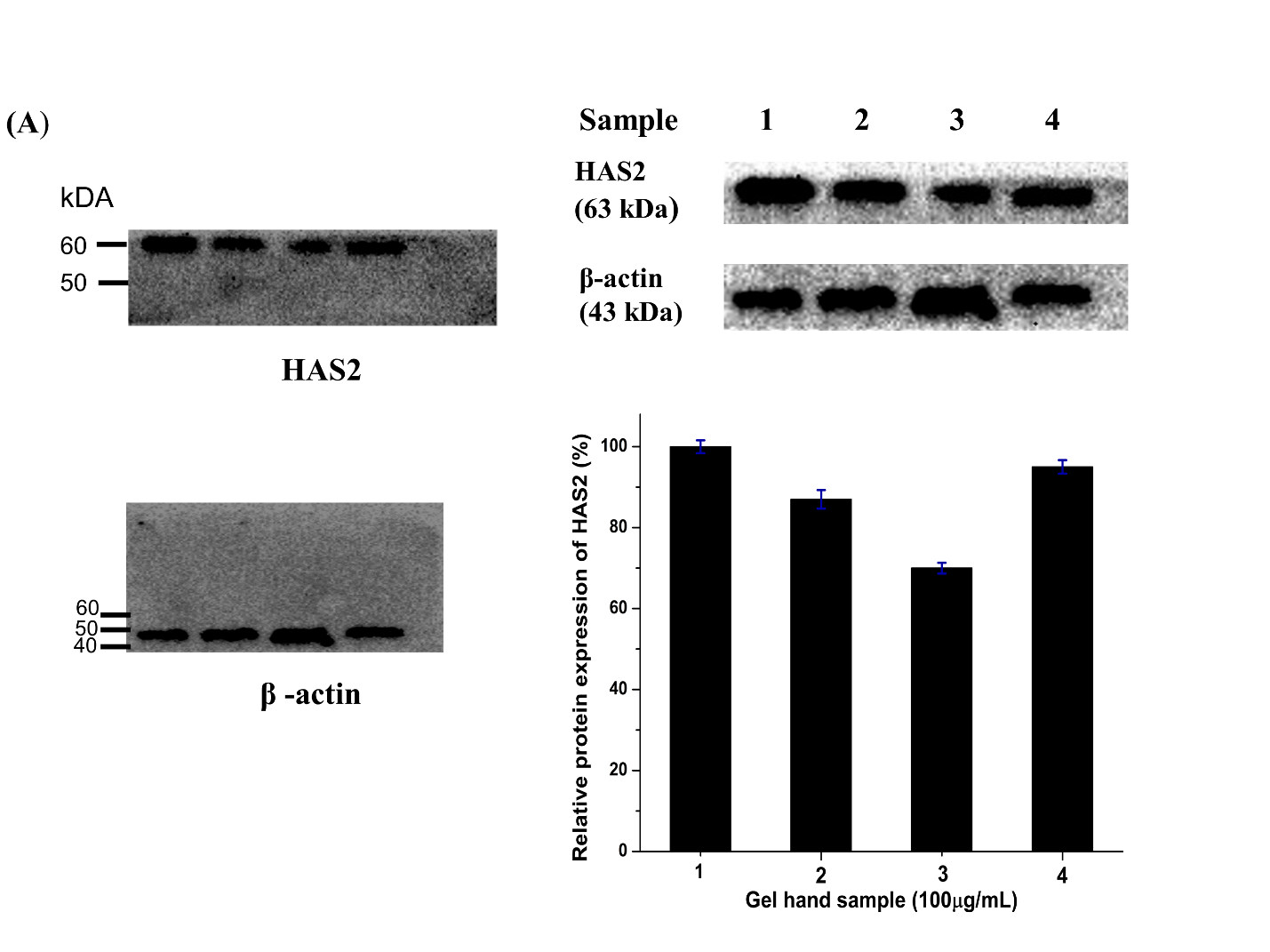
**

**
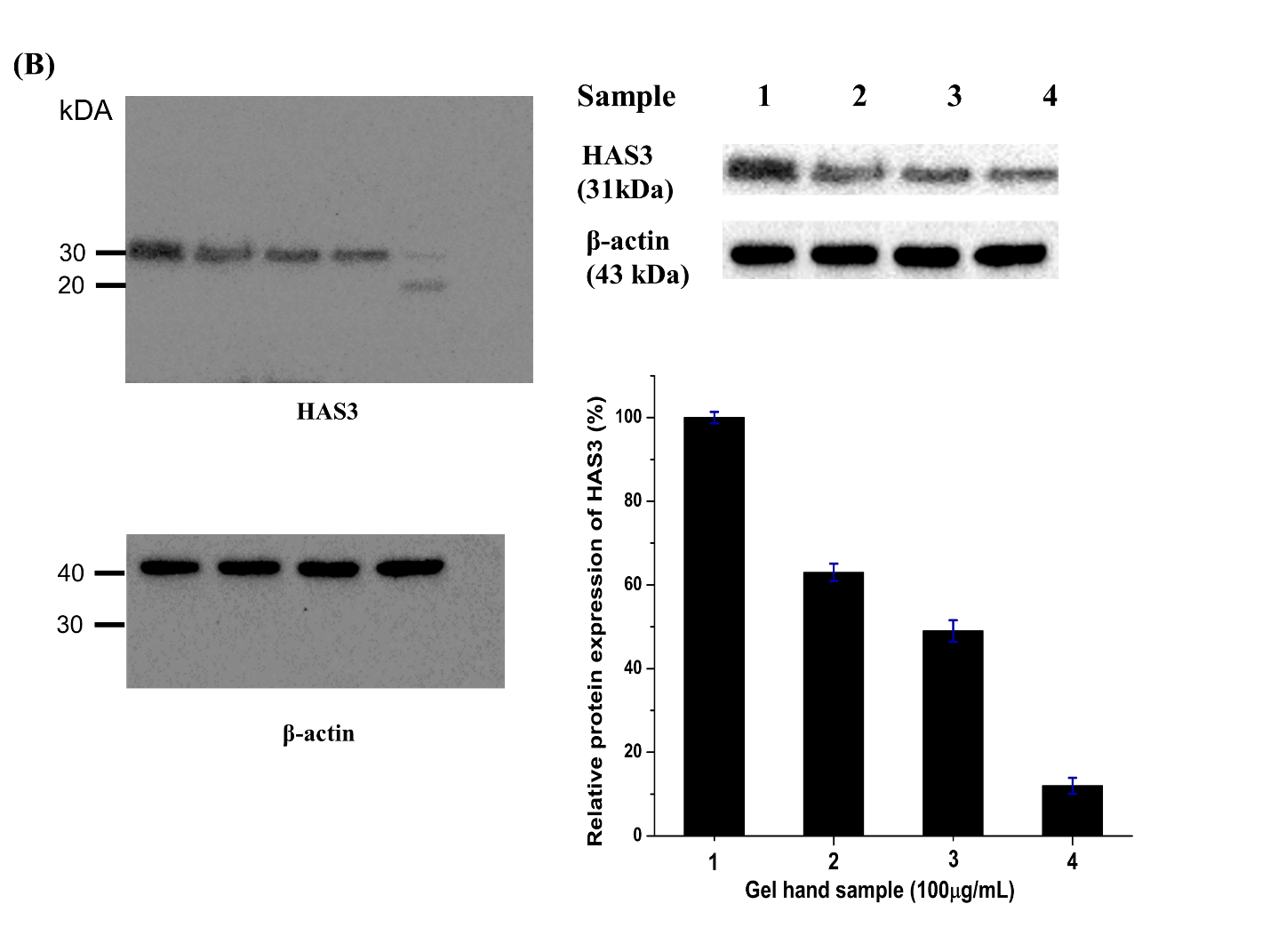
**

**
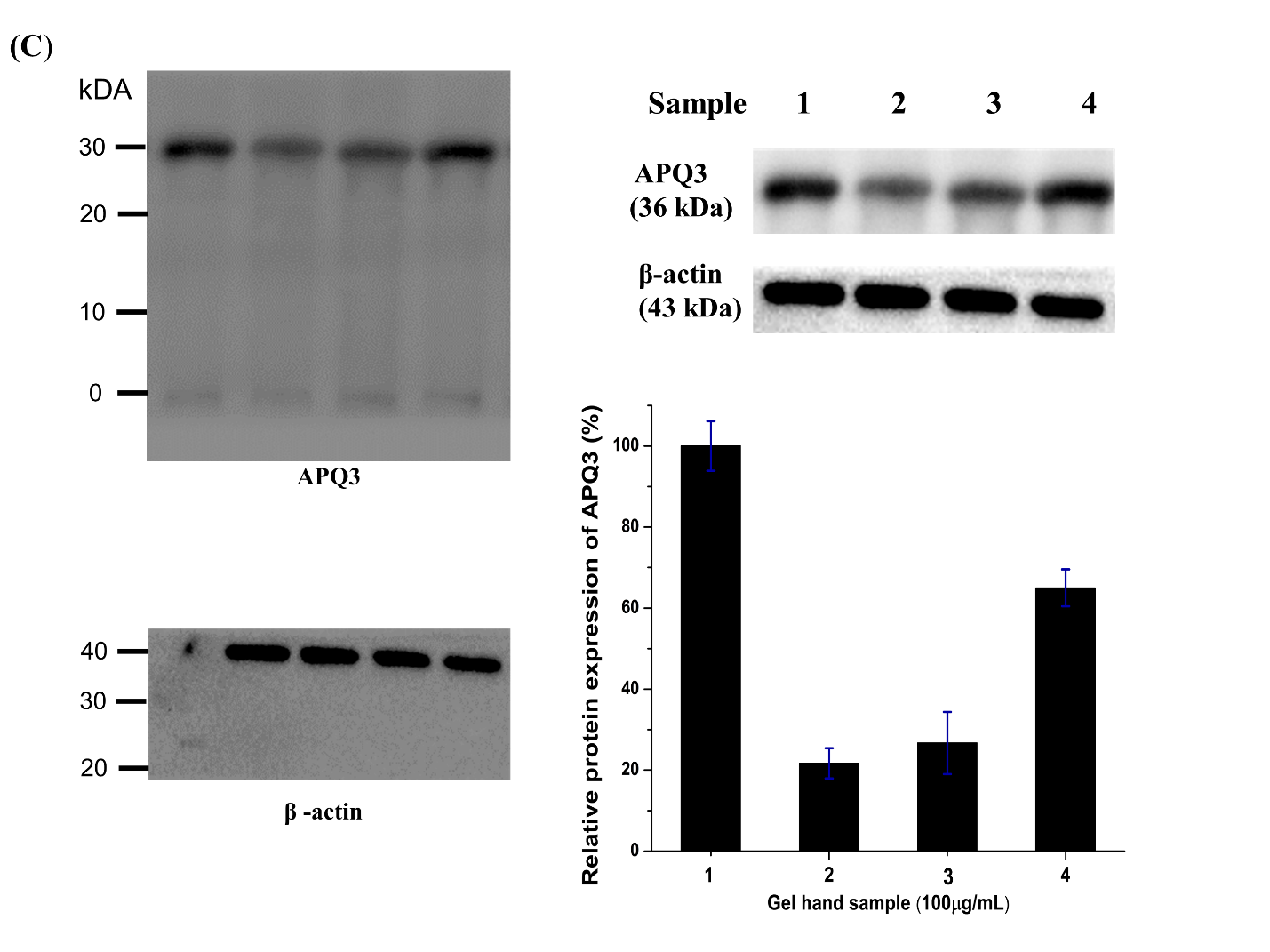
**

**Fig. S4.** Images of the Western blot analysis showed levels of moisturizing-related proteins (HAS2 (A), HAS3 (B), and APQ3 (C)). Blots were probed with specific antibodies, and bands were detected using the secondary antibody. The blots were cut prior to hybridization with antibodies during blotting. The results are expressed as mean ± standard error of mean, n = 3. The relative protein expression data of HAS2, HAS3, and APQ3 were analyzed by Origin8.5.


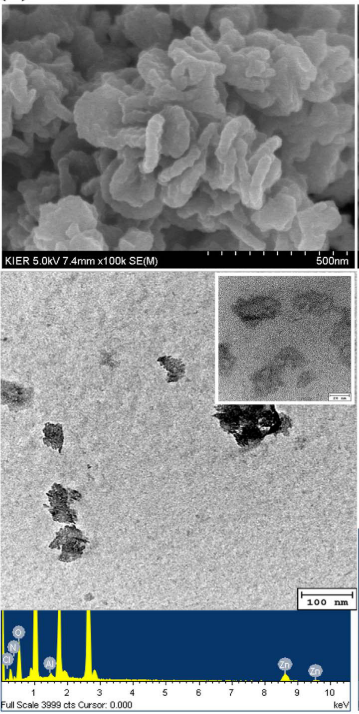


**Fig. S5.** Scanning electron microscope (SEM; top) image, transmission electron microscope (TEM; middle) images, and energy dispersive X-ray analysis (bottom) of ZnAC. Reprinted with permission from Chun et al. (2017)^42^. Copyright 2017, Elsevier.
